# FGF21 Serum Levels in the Early Second Trimester Are Positively Correlated With the Risk of Subsequent Gestational Diabetes Mellitus: A Propensity-Matched Nested Case-Control Study

**DOI:** 10.3389/fendo.2021.630287

**Published:** 2021-04-28

**Authors:** Zhiheng Wang, Min Yuan, Chengjie Xu, Yang Zhang, Chunmei Ying, Xirong Xiao

**Affiliations:** ^1^ Clinical Laboratory, Obstetrics and Gynecology Hospital of Fudan University, Shanghai, China; ^2^ Department of Obstetrics, Obstetrics and Gynecology Hospital of Fudan University, Shanghai, China; ^3^ Information Section, Obstetrics and Gynecology Hospital of Fudan University, Shanghai, China

**Keywords:** FGF21, gestational diabetes mellitus (GDM), nested case-control study, propensity score matching (PSM), early second trimester

## Abstract

**Background:**

As an important endocrine hormone regulating glucose metabolism, fibroblast growth factor 21 (FGF21) is increased in individuals with gestational diabetes mellitus (GDM) after 24 gestational weeks. However, it is unknown whether the increase in FGF21 precedes the diagnosis of GDM.

**Methods:**

In this nested case-control study, 133 pregnant women with GDM and 133 pregnant women with normal glucose tolerance (NGT) were identified through propensity score matching, and serum FGF21 levels were measured at 14 to 21 gestational weeks, before GDM is routinely identified. The differences in FGF21 levels were compared. The association between FGF21 and the occurrence of GDM was evaluated using logistic regression models with adjustment for confounders.

**Results:**

The serum FGF21 levels of the GDM group at 14 to 21 gestational weeks were significantly higher than those of the NGT group overall (*P* < 0.001), with similar results observed between the corresponding BMI subgroups (*P* < 0.05). The 2nd (OR 1.224, 95% CI 0.603–2.485), 3rd (OR 2.478, 1.229–5.000), and 4th (OR 3.419, 95% CI 1.626–7.188) FGF21 quartiles were associated with greater odds of GDM occurrence than the 1st quartile after multivariable adjustments.

**Conclusions:**

The serum FGF21 levels in GDM groups increased in the early second trimester, regardless of whether participants were stratified according to BMI. After adjusting for confounding factors, the FGF21 levels in the highest quartile were associated with more than three times higher probability of the diagnosis of GDM in the pregnancy as compared to levels in the first quartile.

## Introduction

Gestational diabetes mellitus (GDM), defined as glucose intolerance during pregnancy, has an incidence of 7% to 17.5% among pregnant women in China (1–4). GDM can adversely affect mothers and fetuses, resulting in an increased risk of delivery-related complications and type 2 diabetes mellitus (T2DM) (3, 5, 6). Appropriate intervention in pregnant women with GDM can reduce the risk of adverse pregnancy outcomes (7–10). Early diagnosis of GDM before 24 weeks of gestation may provide more time for pregnant women with high risk of GDM for appropriate intervention. However, early identification of GDM is very difficult (2, 11), as GDM is a complex metabolic process of insulin resistance and islet β cell proliferation disorders (2, 12), with many changes in metabolic factors preceding hyperglycemia (13, 14).

Fibroblast growth factor 21 (FGF21), an important endocrine factor regulating glucose and lipid metabolism (15–17), may be a potential factor associated with GDM prediction, as it was recently found that serum FGF21 increased after 24 gestational weeks in GDM patients (18–20). Studies showed that FGF21 reduces blood glucose and regulates blood lipids without causing hypoglycemia in obese or type 2 diabetes patients (21–23), and FGF21 increases insulin sensitivity and improves islet β cell secretion and proliferation (15, 22, 23). As a metabolic factor, FGF21 increases in type 2 diabetes and obesity patients (23, 24), which might be a kind of compensation for insulin deficiency (25) and one study confirmed that serum FGF21 increased before type 2 diabetes was diagnosed in women (26). Due to FGF21 increasing in both GDM and T2DM and the similarity of the pathogenesis of these two diseases (2), we hypothesized that increases in FGF21 compensate for GDM. FGF21 may increase earlier than we are generally aware, and this increase may precede the hyperglycemia of GDM. However, to the best of our knowledge, no study has focused on the changes in FGF21 before the diagnosis of GDM.

Therefore, we intend to analyze the difference in serum levels of FGF21 between individuals with GDM and those without GDM early in the second trimester and analyze the relationship between FGF21 and GDM through a nested case-control study by propensity score matching to eliminate confounding factors.

## Materials and Methods

### Study Population

This study was a nested case-control design carried out by screening the Down’s syndrome screening cohort (14–21 gestational weeks) between January 2019 and October 2019 and was approved by the Ethics Committee of Obstetrics and Gynecology Hospital of Fudan University. All participants were informed of the purpose of the study and signed informed consent forms. Among the cohort of 2,540 participants, 1,368 pregnant women signed informed consent forms for our study.

The inclusion criteria: women with a singleton pregnancy in the Down’s syndrome screening cohort (14–21 gestational weeks) and records of oral glucose test (OGTT) at 24 to 28 weeks of pregnancy. A total of 1247 pregnant women who met the inclusion criteria were included in the study. Individuals with any of the following were excluded: no GDM diagnostic information; pregnancy with twins or triplets; diabetes history; malignant tumors; serious metabolic diseases including Cushing’s syndrome, hyperthyroidism and hypothyroidism; or severe hypertension.

One hundred forty subjects were diagnosed with GDM by a 75-g oral glucose test (OGTT) at 24 to 28 weeks of pregnancy according to the Diagnostic Criteria for Gestational Diabetes issued by the International Association of Diabetes and Pregnancy Study Groups (27, 28). GDM was diagnosed if any one of the following was met: (1) fasting plasma glucose ≥5.1 mmol/L; (2) 1-h plasma glucose ≥10 mmol/L; or (3) 2-h plasma glucose ≥8.5 mmol/L.

**Figure 1:**
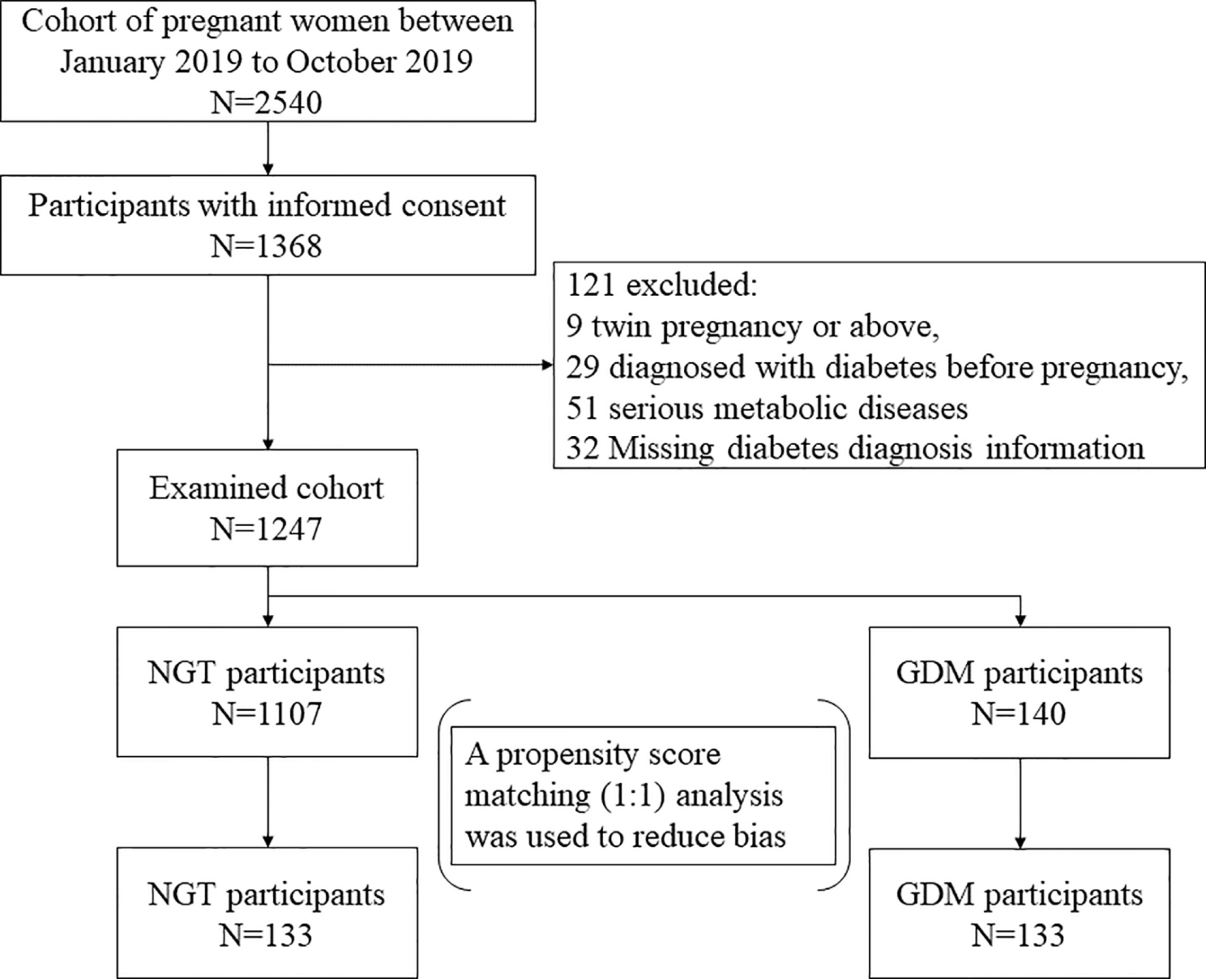
The flowchart of the selection of the analyzed GDM population and the matched control population.

Finally, 133 individuals with GDM and 133 individuals with normal glucose tolerance (NGT) were identified as our research subjects through propensity score matching. In the process of object matching, the following multiple covariates and potential confounding factors associated with GDM were considered: age, body mass index (BMI) early in the second trimester, ethnicity, and the number of parities. Since all GDM subjects were Han Chinese and the number of parities is similar (zero or one) in Chinese women, age and BMI were finally determined as matching variables. The GDM and NGT pregnant women were matched in a 1:1 ratio using the nearest neighbor algorithm (caliper width 0.04 of the SD for the logit propensity score) without replacement. The flowchart of the selection of the analyzed GDM population and the matched control population is shown in **Figure 1**.

Considering that BMI is an important factor that affects FGF21 levels and the occurrence of GDM (29–32), comparative analyses of the BMI subgroups were performed in this study. Participants were grouped on the basis of the Chinese BMI classification standard into underweight (BMI < 18.5 kg/m^2^), normal weight (18.5 ≤ BMI < 24 kg/m^2^), overweight (24 ≤ BMI < 28 kg/m^2^) and obese (BMI ≥ 28 kg/m^2^) subgroups (33).

### Detection of Serum FGF21

Serum was collected during Down’s syndrome screening (14–21 gestational weeks) and stored at −20°C. The serum levels of FGF21 were detected by an Abcam ELISA kit (ab222506, Cambridge, UK).

### Statistical Analysis

All statistics were performed by SPSS software. Psmatch 3.04 of the R language extender in SPSS software (Version 25.0; IBM, NY, US) was used for propensity score matching. The results are expressed as the mean ± standard deviation if the data followed a normal distribution, while abnormally distributed measurements are reported as the median (interquartile range). The t-test, Mann-Whitney U test, Kruskal-Wallis one-way ANOVA test, chi-square test, or Fisher’s exact test was used to compare the differences in variables among groups as required. All reported P values were two-tailed. Binary logistic regression analysis models were used to estimate the associations of FGF21 with GDM. Model 1 was adjusted for the known risk factors for GDM, including age, BMI, family history of metabolic diseases and parity. *P* < 0.05 was considered statistically significant.

## Results

To verify the effect of eliminating confounding factors after sample matching, the age, BMI, gestational weeks at blood sampling, gestational weeks at OGTT, family history of metabolic diseases and parity in the overall GDM and NGT groups and each corresponding subgroup were compared, and the results are reported in **Table 1**. It should be noted that statistical analysis between underweight subgroups was not performed due to the small number of cases (2 of GDM, 1 of NGT). As expected, there was no significant difference in age, BMI, gestational age, family history of metabolic diseases or parity between the GDM and NGT groups, regardless of whether analyzed overall or by corresponding subgroup (*P*>0.05, **Table 1**), which suggested that the effect of confounding factors was sufficiently reduced after matching.

**Table 1:** Baseline characteristics of the study population and the serum levels of FGF21 in different groups.

		NGT	GDM	*P* value
Total				
	N	133	133	NA
	Age (y)	29.54 ± 2.62	29.53 ± 2.27	0.972
	BMI (kg/m^2^)	22.50 (20.85–24.21)	22.80 (20.70–24.50)	0.704
	Gestational weeks at blood sampling (w)	16.39 ± 0.92	16.48 ± 0.79	0.393
	Gestational weeks at OGTT (w)	25.93 ± 1.36	25.63 ± 1.25	0.339
	Family history of metabolic diseases (N)	22	30	0.216
	Parity			0.360
	0	109(82.0)	103(77.4)	
	1	24(18.0)	30(22.6)	
	FGF21 (pg/ml)	24.20 (23.91–67.89)	58.59 (33.34–105.03)**	<0.001
BMI (kg/m^2^) ≥28				
	N	9	9	NA
	Age (y)	28.67 ± 1.73	28.70 ± 1.65	0.967
	BMI (kg/m2)	29.73 (28.68–33.59)	30.70 (29.60–32.65)	0.730
	Gestational weeks at blood sampling (w)	16.00 ± 1.12	16.44 ± 0.73	0.332
	Family history of metabolic diseases (N)	4	2	0.620
	Parity			1.000
	0	8(88.9)	7(77.8)	
	1	1(11.1)	2(22.2)	
	FGF21 (pg/ml)	33.35 (30.60–103.90)	177.42 (88.89–307.22)*	0.011
24≤BMI (kg/m^2^) <28				
	N	28	32	NA
	Age (y)	29.75 ± 2.96	29.42 ± 2.19	0.625
	BMI (kg/m^2^)	25.48 (24.50–26.40)	25.40 (24.50–26.38)	0.935
	Gestational weeks at blood sampling (w)	16.57 ± 1.03	16.63 ± 0.83	0.825
	Family history of metabolic diseases (N)	5	13	0.102
	Parity			0.165
	0	21(75)	29(90.6)	
	1	7(25)	3(9.4)	
	FGF21 (pg/ml)	44.20 (27.38–109.83)	75.43 (43.52–159.17)*	0.042
18.5≤BMI (kg/m^2^) <24				
	N	95	90	NA
	Age (y)	29.59 ± 2.59	29.70 ± 2.35	0.804
	BMI (kg/m^2^)	21.58 (20.39–22.86)	21.55 (20.30–23.03)	0.981
	Gestational weeks at blood sampling (w)	16.37 ± 0.86	16.43 ± 0.79	0.621
	Family history of metabolic diseases (N)	13	15	0.572
	Parity			0.073
	0	79(83.2)	65(72.2)	
	1	16(16.8)	25(27.8)	
	FGF21 (pg/ml)	33.36 (21.74–66.78)	51.67 (28.86–87.49)**	0.005

Data are presented as the mean ± standard deviation, median (interquartile range) or N (%). Data on age and gestational weeks at blood sampling were compared using the t-test; BMI and FGF21 were compared using the Mann-Whitney U test; family history of metabolic diseases and parity were compared using the chi-square test and Fisher’s exact test. *P<0.05 and **P<0.01 compared with the control groups. NGT, normal glucose tolerance group; GDM, gestational diabetes mellitus group; N, number; BMI, body mass index; FGF21, fibroblast growth factor 21, NA, not applicable.

Then, the differences in serum levels of FGF21 among the corresponding groups were analyzed (**Table 1**). The FGF21 levels of the GDM group [58.59 (33.34–105.03) pg/ml] were significantly higher than those of the NGT group [24.20 (23.91–67.89) pg/ml] overall (*P*<0.001), with similar results observed between the corresponding BMI subgroups (all *P*<0.05). In GDM group, the FGF21 levels in the normal BMI subgroup [51.67 (28.86–87.49) pg/ml] were the lowest, followed by the overweight group [75.43 (43.52–159.17) pg/ml], and the levels were highest in the obese group [177.42 (88.89–307.22) pg/ml], with a significant difference between the obese group and the normal BMI group (*P* = 0.002, Bonferroni corrected). However, there was no significant difference among the different BMI NGT subgroups (Bonferroni, *P* = 0.154).

Subsequently, the relationship of FGF21 and GDM was explored using logistic regression analysis by stratifying study subjects into quartiles for FGF21 (Q1 ≤ 27.83 pg/ml, Q2 28.00 to 45.82 pg/ml, Q3 46.81 to 83.87 pg/ml, Q4 ≥84.09 pg/ml; **Table 2**). Compared with Q1, we found that Q3 was associated with a high risk of GDM (adjusted for age, BMI, family history of metabolic diseases and parity) (OR 2.478, 95% CI 1.229–5.000; P = 0.011). Moreover, Q4 was related to a higher risk of GDM (OR 3.419, 95% CI 1.626–7.188; P = 0.001). This demonstrated that FGF21 levels significantly increased early in the second trimester, which was an independent risk factor for GDM.

**Table 2:** Associations of serum FGF21 concentrations (pg/ml) early in the second trimester of pregnancy with the risk of gestational diabetes mellitus.

FGF21 (pg/ml) (quartiles)	NGT (N=133)	GDM (N=133)	Crude OR	adjusted OR^a^
1st Quartile (≤27.83)	43 (32.3%)	24 (18.0%)	1.000 (referent)	1.000 (referent)
2nd Quartile (28.00–45.82)	39 (29.3%)	27 (20.3%)	1.240 (0.616–2.498)	1.224 (0.603–2.485)
3rd Quartile (46.81–83.87)	28 (21.1%)	39 (29.3%)	2.496 (1.244–5.008)	2.478 (1.229–5.000)
4th Quartile (≥84.09)	23 (17.3%)	43 (32.3%)	3.350 (1.645–6.821)	3.419 (1.626–7.188)
*P* for trend	NA	NA	0.002	0.002

Values are given as OR (95% CI).

a Adjusted for age, BMI, family history of metabolic diseases and parity.

OR, odds ratio; CI, confidence interval; NGT, normal glucose tolerance group; GDM, gestational diabetes mellitus group; N, number; BMI, body mass index; FGF21, fibroblast growth factor 21; NA, not applicable.

## Discussion

To the best of our knowledge, this is the first study to evaluate the association between the serum levels of FGF21 and GDM before recommended routine screening period. Through a propensity-matched nested case-control study, we found that the FGF21 levels at 14 to 21 gestational weeks in the GDM groups were significantly higher than those in the NGT groups, regardless of whether analyzed overall or by corresponding BMI subgroup. And higher levels of FGF21 were significantly associated with a higher risk of GDM. Of note, these relationships were independent of known risk factors for GDM, including age, BMI, family history of metabolic diseases and parity.

Due to the important role of FGF21 in regulating glucose metabolism in the body, its potential role in GDM has gradually received increasing attention in recent years. Most of the studies found that the serum levels of FGF21 in GDM women were all elevated after 24 gestational weeks. Li et al. and Bonakdaran et al. found that GDM women had higher levels of FGF21 than NGT pregnant women at 24 to 28 gestational weeks (18, 34). ŠIMJÁK et al. found that FGF21 in GDM women was higher than that in NGT pregnant women at 28 to 32 gestational weeks (35). In addition, four studies showed that FGF21 was also higher in the GDM group than in the NGT group in the third trimester and prenatally (20, 35–37).

Notably, we discovered that FGF21 increased significantly at 14 to 21 gestational weeks before the routine GDM diagnosis time (24–28 gestational weeks). The impaired glucose tolerance on pregnancy is a continuum from normal to established GDM (38). It is possible that glucose tolerance of GDM women is impaired right at the outset (even outside of pregnancy) than that of non-GDM women. It may be why FGF21 levels in women who later were diagnosed with GDM were higher than those with normal OGTT. Our findings might make it possible for considering FGF21 as a predictor of GDM. Moreover, GDM women with normal BMI account for more than half of all GDM women in China (1, 2, 30, 39), and the possibility of GDM onset in women with normal BMI is more likely to be ignored and difficult to predict. Importantly, we found that the FGF21 levels of the normal BMI GDM subgroup were significantly higher than that of the normal BMI NGT subgroup, which provided important theoretical evidence for the early prediction of GDM in normal BMI pregnant individuals. Prospective studies are needed to confirm the predictive value of FGF21 for identifying the women at high risk of GDM.

In addition, our study showed that higher levels of FGF21 were associated with a higher risk of GDM. Our findings raise an important question regarding the potential mechanism or causality for the development of GDM with elevation of FGF21. As studies have found that FGF21 can improve tissue insulin sensitivity, promote the proliferation of pancreatic β-cells, and increase insulin secretion (15, 22, 23, 25), we suspected that there was a compensatory effect of FGF21 in pregnant women before GDM was identified (25). In addition, studies found that there were some differences in pathogenesis between obese and nonobese GDM women (2, 33, 40), and in our study, the FGF21 levels of the obese GDM subgroup were significantly higher than those of normal BMI GDM subgroup, which suggested that the mechanism of FGF21 resistance might be different in GDM women with different BMIs. Therefore, interventions that influence FGF21 levels based on different BMIs should be focused on for future research to prevent subsequent GDM.

Though high-quality results were demonstrated in our study by eliminating the effects of BMI and age through propensity score matching in a large cohort, there were still limitations. This was a retrospective study carried out by a Down’s syndrome screening cohort, which lacked some important information, such as real-time blood glucose and glycosylated hemoglobin levels; therefore, the hyperglycemia and insulin resistance status of the individuals included were unknown. Therefore, it was not known whether some individuals already had hyperglycemia at 14 to 21 weeks of gestation, which brought a certain degree of uncertainty in the clinical value of the availability of high FGF21 levels. Nevertheless, our results still showed that higher levels of FGF21 were significantly associated with higher detection rate of GDM, which could provide evidence for the earlier identification of women at high risk of GDM before the recommended GDM screening period. Prospective studies are needed to confirm the clinical value of FGF21 for identifying the women at high risk of GDM. Another limitation was that the pregnant women in this study cohort were generally younger than 35 years old; therefore, research on FGF21 in GDM women over 35 years old still needs to be performed. However, studies have shown that pregnant women younger than 35 years old account for more than 85% of the total number of pregnant women in China (41). Therefore, the findings of this study are still very instructive and interesting.

In summary, the serum FGF21 levels in GDM women increased early in the second trimester, regardless of whether participants were stratified according to BMI. After adjusting for confounding factors, the FGF21 levels in the highest quartile were associated with more than three times higher probability of the diagnosis of GDM in the pregnancy as compared to levels in the first quartile. In addition to the currently established clinical and biochemical risk factors, the circulating concentration of FGF21 represents a potentially useful new biomarker that can identify pregnant women at risk for GDM.

## Data Availability Statement

The data that support the findings of this study are available on request from the corresponding author. The data are not publicly available due to privacy or ethical restrictions. Requests to access these data sets should be directed to ZW, wzh.0409@163.com.

## Ethics Statement

The studies involving human participants were reviewed and approved by the ethics committee of Obstetrics and Gynecology Hospital of Fudan University. The patients/participants provided their written informed consent to participate in this study.

## Author Contributions

XX, CY, and ZW contributed to the conception of the study. ZW, CX, and YZ contributed significantly to analysis and manuscript preparation. ZW and MY performed the data analyses and wrote the manuscript. XX and ZW helped perform the analysis with constructive discussions. All authors contributed to the article and approved the submitted version.

## Funding

This work was partly supported by the Shanghai Municipal Health Bureau (grant 20204Y0404) and National Natural Science Foundation of China (81871183), and Shanghai Committee of Science and Technology (18411963400).

## Conflict of Interest

The authors declare that the research was conducted in the absence of any commercial or financial relationships that could be construed as a potential conflict of interest.
